# A New Way of Assessing Foraging Behaviour at the Individual Level Using Faeces Marking and Satellite Telemetry

**DOI:** 10.1371/journal.pone.0049719

**Published:** 2012-11-16

**Authors:** Marie-Andrée Giroux, Christian Dussault, Nicolas Lecomte, Jean-Pierre Tremblay, Steeve D. Côté

**Affiliations:** 1 NSERC-Produits Forestiers Anticosti Industrial Research Chair, Département de Biologie and Centre d’Études Nordiques, Université Laval, Québec, Québec, Canada; 2 Direction Générale de l’Expertise sur la Faune et ses Habitats, Ministère des Ressources Naturelles et de la Faune, Québec, Québec, Canada; 3 Département de Biologie, Université du Québec à Rimouski, Rimouski, Québec, Canada; 4 Department of Environment, Government of Nunavut, Igloolik, Nunavut, Canada; Hokkaido University, Japan

## Abstract

Heterogeneity in foraging behaviour can profoundly influence ecological processes shaping populations. To scale-up from individual foraging behaviour to processes occurring at the population scale, one needs to sample foraging behaviour at the individual level, and over large temporal scales or during critical seasons known to influence life-history traits. We developed an innovative technique to monitor foraging behaviour at the individual level in secretive species, a technique that can be ultimately used to investigate the links between foraging behaviour and life-history traits. First, the technique used a novel approach, namely the combination of telemetry tracking and biomarking of faeces with food dyes to locate fresh signs of presence left by individuals equipped with GPS collars. Second, the technique is based on the simultaneous or successive sampling of life-history traits and individual foraging behaviour, using tracks with high probabilities of recovery of dyed faeces. We first describe our methodological approach, using a case study of a large herbivore, and then provide recommendations and guidelines for its use. Sampling single snow tracks of individuals equipped with a GPS collar was a reliable way to assess individual winter foraging behaviour in a white-tailed deer (*Odocoileus virginianus* Zimmermann) population. During that period, the probability of recovery of dyed faeces within the range of the collar precision was very high for single snow tracks of equipped deer (97%). Our approach is well suited to study individual foraging behaviour, and could ultimately be used to investigate the interplay between intra-population heterogeneity in foraging behaviour, life-history traits, and demographic processes.

## Introduction

Individual variation in foraging behaviour can profoundly influence ecological processes shaping populations [1]. Inter-individual variation in foraging behaviour within populations may, for instance, reduce intraspecific competition [2], [3] and modulate the risk of predation and parasitism [4]. Such heterogeneity can be a strong determinant of life-history traits [5], [6], [7], thus shaping demographic processes underlying population dynamics [8], and recent years have seen a surge in studies focusing on the importance of inter-individual variations at various temporal scales (e.g. [9], [10], [11]).

To scale-up from individual foraging behaviour to ecological processes occurring at the population scale, one first needs to link individual foraging behaviour with life-history traits. This requires the sampling of foraging behaviour at the individual level, but also over a large temporal scale or during critical seasons known to influence life-history traits. Sampling techniques have been developed to investigate foraging behaviour at the individual level, yet several constraints can limit their applicability in specific study systems, for instance in the study of secretive species or for investigating processes over large temporal scales [12], [13]. We developed a technique to monitor individual foraging behaviour over variable temporal scales in secretive species, a technique that could be ultimately used to investigate the influence of foraging behaviour on life-history traits. First, our technique relies on a novel approach, namely the combination of telemetry tracking and biomarking of faeces with food dyes to sample fresh signs of presence left by individuals equipped with GPS collars. Second, the technique is also based on sampling life-history traits simultaneously or successively to individual foraging behaviour, using tracks with high probabilities of recovery of dyed faeces. We here describe the methodological approach we developed, using a case study of a large herbivore population, and then provide guidelines for determining whether it could be used in a given study system and which type of questions could be investigated.

## Methods

### Ethics Statement

All animals handling protocols were approved by the Université Laval Animal Care Committee of the Canadian Council on Animal Care (Permit number: UL 2008017-2). To conduct field work on Anticosti Island, we also obtained an authorisation for scientific studies from the Ministère des Ressources naturelles et de la Faune du Québec (Permit number: 09052701409SF).

### Methodological Approach

**Figure 1:**
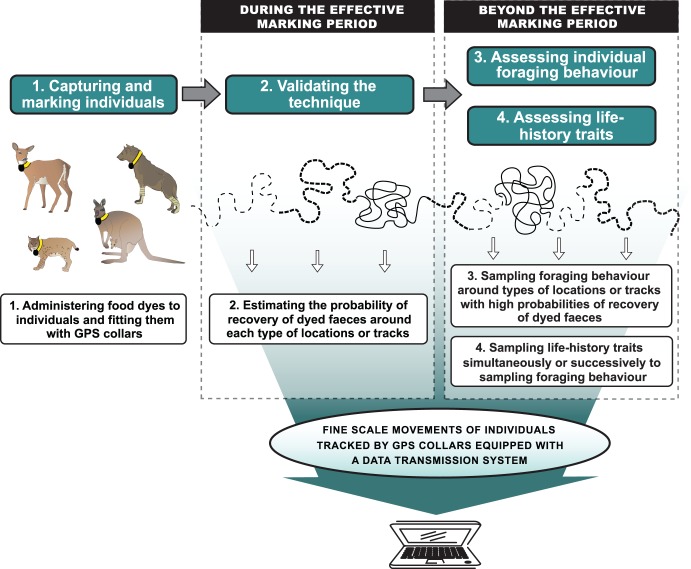
Schematic representation of the four-step approach to sample foraging behaviour at the individual level using faeces marking with food dyes and GPS telemetry.

To verify the reliability of GPS telemetry to sample foraging behaviour of marked individuals throughout winter, we administred food dyes orally to mark individuals’ faeces. We subsequently visited in the field recent GPS locations of collared animals received via a data transmission system (satellite, GSM or radio transmission system) to estimate the probability of recovery of dyed faeces near different types of locations or tracks. Our proximate goal was to be able to use this technique beyond the effective marking period, i.e. when the dye is no longer effective, to sample foraging behaviour near the types of locations or tracks with high probabilities of recovery of dyed faeces (hereafter “the validated types of locations or tracks”). Our ultimate goal was to assess life-history traits of the collared individuals simultaneously or successively to individual foraging behaviour in order to establish the links between those two concepts. This technique used the four steps illustrated in Figure 1. We describe below these four steps, using the case study of a white-tailed deer (*Odocoileus virginianus* Zimmermann) population on Anticosti Island (Quebec, Canada, 49.0–49.9°N, 61.6–64.5°W; 7,943 km^2^) to illustrate how the technique can be used in winter. Deer on Anticosti Island do not gather in deer yards during winter and single snow tracks are frequently encountered [14].

#### First step – Capturing and marking individuals

We captured 15 and 12 female deer during winters 2008–2009 and 2009–2010, respectively, using Stephenson box traps, cannon nets and net guns fired from a helicopter. We anesthetized deer by injecting a mixture of Telazol (Fort Dodge Animal Health, Fort Dodge, USA) and Xylazine (Bimeda-MTC Animal Health Inc., Cambridge, Canada) at doses of 3.2 mg/kg and 1.6 mg/kg, respectively [15], administered with a syringe following physical contention of the animal. After chemical immobilization, we fitted deer with GPS collars equipped with an Iridium satellite transmission device allowing the frequent reception of locations (Vectronic-Aerospace, Berlin, Germany).

To mark their faeces, each deer was given 5 to 6 gelatine capsules (5 cc) each containing 2 g of blue food dyes in powder (FD&C Blue No. 1, also called Brilliant Blue FCF; International Foodcraft Corporation, Linden, USA). Capsules were administered orally into the oesophagus with a dosing-gun (Cotran Corporation, Portsmouth, USA). The choice of FD&C Blue No. 1 was based on a captivity experiment aiming to test the effectiveness of the three following dyes at staining faeces: Beet Juice Powder, FD&C Blue No. 1 and FD&C Blue No. 2 (International Foodcraft Corporation, Linden, USA). Among those three dyes, FD&C Blue No. 1 was the only one providing an easily detectable stain of the faeces on the forest floor or snow. To avoid marking deer with overlapping home ranges, we captured deer at >2 km from one another (the maximal length of winter home range in this study population was 1.8 km; Massé and Côté, unpublished results). Prior to release, we administered Yohimbine (Lloyd Laboratories, Shenandoah, USA), an antagonist to Xylazine, at doses of 0.2 mg/kg [16].

#### Second step – Validating the telemetry technique with dyed faeces

**Figure 2:**
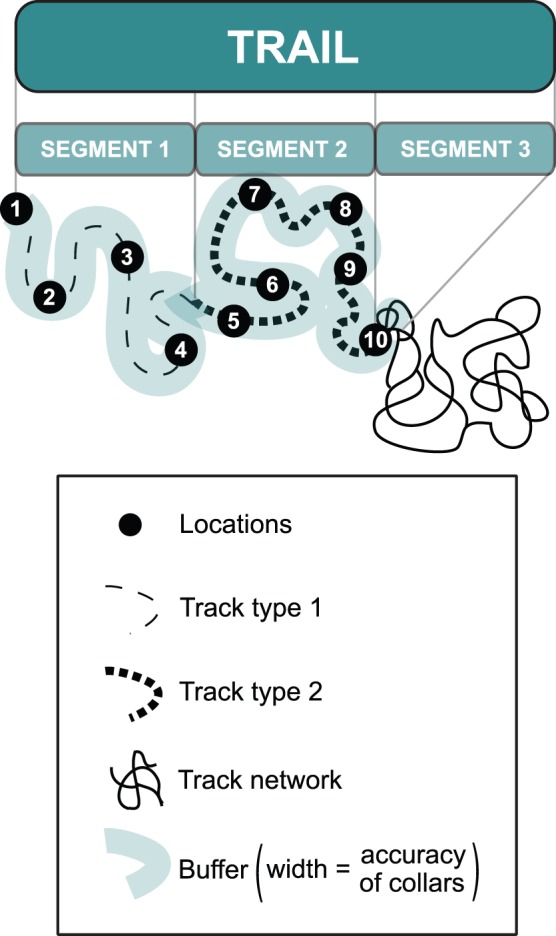
Schematic representation of a trail as defined in our methodological approach. The trail is formed by tracks linking successive GPS locations recorded frequently (e.g. every five minutes in our study of a white-tailed deer population). The trail is divided into different segments. Each segment is characterised by a single type of tracks (including a buffer on each side of the track, with buffer’s width corresponding to the average accuracy of the GPS collar). In the case of a network of tracks, a segment corresponds to the buffer around the beginning of the network.

We quantified the probability of recovery of dyed faeces in the snow tracks found in the vicinity of deer locations transmitted by the Iridium-GPS satellite collars. For each collared deer, we recorded one telemetry location every five minutes between 4∶00 and 20∶00 daily; we chose this schedule because it encompassed day and dusk, i.e. the two periods of greatest deer activity during winter in this population [17]. We transferred all telemetry locations received by email to a handheld GPS (Garmin eTrex, Southampton, UK) and visited locations shortly after their satellite transmission (mean time interval between data recordings and field visits: 22 hours, range: 5 hours to 36 hours). Trails sampled in the field corresponded to the tracks formed by linking successive locations recorded every five minutes (Figure 2) over a period varying between 1 and 12 hours (mean = 6 hours, 95% CI = [5], [7]) up to 6 days after dye administration.

At each visited GPS-location along a trail, we classified deer tracks found within a 5 m-radius around the location in the following, mutually exclusive categories: single track (ST), track network (N, i.e. two or more single tracks found within the 5 m-radius), and path (P, i.e. tracks used more than once). The 5 m-radius corresponds to the average accuracy of collar locations (4.3 m, 95% CI = [1.7, 6.9]). We estimated the accuracy of collars using field trials, measuring the average distance between locations recorded by stationary paired GPS collars and handheld GPSs in both open and forest habitats [18]. Accuracy estimates were similar in these two habitats types (see Methods S1 in the Supporting Information for details). We followed snow tracks linking two successive locations and classified tracks as ST, N or P. Each trail was subdivided into segments and each segment was characterised by a single type of tracks (including a 5-m buffer on each side of the track; Figure 2). For track networks, a segment was rather defined as the 5-m buffer around the starting point of the network, as tracks forming a network ran in different directions that did not lead directly to subsequent locations on the trail.

To estimate the probability of recovery of dyed faeces during the effective marking period for all track types, we counted the number of dyed and undyed faeces on each trail segment. For each trail sampled, we noted the occurrence of snowfall between the time of GPS locations and sampling in the field to test for an effect of snowfall on the probability of detection. We only sampled trails following light snowfalls (approximately<3–4 cm) because we could not detect trails after heavier snowfalls (>4 cm). We performed the validation from December 11^th^ to January 12^th^ in 2008–2009, and from February 10^th^ to 16^th^ in 2009–2010.

To determine the probability of recovery of dyed faeces for all types of snow tracks, we fitted mixed logistic regression models with the number of dyed and undyed pellet groups as a binomial response (logit link). All models included the type of tracks and trail ID as fixed and random effects, respectively. To take into account variability in the detection rate of dyed faeces vs. undyed ones, we also modelled the effects of the occurrence of snowfall between the recording of locations and their sampling, the time of sampling during the day, as well as the interactions between track types and these two variables. To test whether the probability of detecting dyed faeces decreased with time elapsed since capture (i.e. to determine if we sampled beyond the effective marking period), we used the time interval between the capture and the location as a covariate. In this analysis, we excluded all sampled segments without faeces, i.e. 115 segments excluded over a total of 211 sampled (a large majority of segments were shorter than 100 m: average length of segments = 79 m, 95% CI = [55,103]). We then built 19 biologically relevant candidate models that could explain variations in the probability of recovering only dyed faeces (see Table S1 in Supporting Information for the full list of models). We used the Akaike information criterion corrected for small sample sizes (AICc) to select the mixed logistic regression model that received the highest empirical support. Models were fitted using maximum likelihood estimation, and final parameters were estimated using the restricted maximum likelihood [20]. We used the model with the smallest AICc to estimate parameter values [21], and we assessed statistical significance using their 95% confidence intervals [22]. We assessed goodness-of-fit with the Pearson’s Chi-Square test. We carried out all statistical analyses with R 2.11.1 [23], using the function glmer in package lme4 [24].

#### Third step – Assessing individual foraging behaviour beyond the effective marking period

We here briefly described the third step of the technique, as well as the fourth step below, to allow a better understanding of the full potential of our technique. Yet, in this study, we will not report results obtained from these two steps. To describe individual diet, we repeatedly visited trails (as defined in the “*Second step*” section above) of individuals equipped with GPS collars throughout winter, and we collected faeces on validated types of tracks (see Results) found along those trails. Second, we assessed fine-scale resource selection by comparing the abundance of food resources on both validated types of tracks used by a given individual and paired transects of available habitat running parallel to those tracks (located within 15–30 m from those tracks). Third, we determined how movement rate of individuals was influenced by the abundance of food resources on the validated types of tracks, using GPS locations to estimate movement rates.

#### Fourth step – Assessing life-history traits simultaneously or successively to sampling foraging behaviour

We sampled critical life-history traits of large temperate herbivores such as winter survival, and female and foetus body mass at the onset of spring [25], [26], [27]. We detected winter mortality simultaneously to sampling foraging behaviour by 1- checking movement patterns of individuals equipped with GPS collars weekly, and 2- locating carcasses of individuals that seemed stationary for more than a few days in a row. We sampled female and foetus body mass at the onset of spring by culling females equipped with collars at the end of the study period, i.e. after having sampled their respective winter foraging behaviour.

## Results

Blue dyes appeared in the faeces of female white-tailed deer as soon as 20 h after ingestion. Across all track types, the probability of recovery of dyed faeces (number of dyed faeces/total number of faeces found on a given segment with faeces) averaged 0.44 (95% CI = [0.35,0.59], n = 98). Track type best explained variations in the probability of recovery of dyed faeces (best-fitting model: track type as a single fixed effect, see Table S1 for model selection). According to the best-fitting model, the predicted probability of recovery of dyed faeces for single tracks was very high (0.97, 95% CI = [0.90,1.00], n = 13, Figure S1) compared to other track types (networks: 0.33, 95% CI = [0.19,0.48], n = 73; paths: 0.15, 95% CI = [0.04,0.47], n = 11). Our results also confirmed that we sampled all trails within the effective marking period because the probability of recovery of dyed faeces did not vary with time elapsed since animal capture (slope = 6% per day, 95% CI = [-36%, 74%]; according to the model including the additive effect of time elapsed since animal capture and track types, see Table S1). In our study, the maximal time interval between any capture and trail sampling was 6 days, so the effective marking period was at least 6 days. Adding occurrence of snowfall and time of sampling during the day to the model with track types did not improve model fit (see Table S1).

## Discussion

We developed and described an innovative technique for sampling foraging behaviour at the individual level in secretive species over variable temporal scales. This technique is based on a combination of telemetry tracking of animal movements and biomarking. The novelty in our approach is that we fitted individuals with GPS collars equipped with a data transmission system and marked their faeces with food dyes effective during a short-term period; this allowed the identification of tracks exclusively used by equipped individuals within the range of the GPS collar accuracy. This technique allows to sample individual foraging behaviour with high certainty on validated tracks, and could ultimately allow the determination of how foraging behaviour influences life-history traits.

To discuss the implications of this technique, we will address the three following points: 1- Linking foraging behaviour with life-history traits, 2- add-ons to existing methods of sampling foraging behaviour, and 3- guidelines to determine whether this technique can be used in a given study system and which type of questions could be investigated.

### Linking Foraging Behaviour with Life-history Traits

Quantification of individual foraging behaviour is an essential step to link foraging behaviour with life-history traits, provided that: 1- such quantification is performed over a temporal scale long enough to capture its influence on life-history traits, and 2- those traits can be estimated simultaneously or succesively to the sampling of foraging behaviour. The novel component of our technique was designed to assess individual foraging behaviour beyond the effective marking period (Figure 1) over seasonal or larger temporal scales in secretive species. Indeed, one can confidently assess the foraging behaviour of any particular individual equipped with a GPS collar as long as the validated type of tracks or locations can be found, and GPS collars have enough battery power to record and transmit data. In our study, description of individual foraging behaviour was possible throughout the winter or as long as we could find single snow tracks. From the validated type of tracks or locations, we described individual diet by repeatedly collecting faeces, quantified individual food resource selection by assessing the availability and use of different food resources, and estimated animal movement rate in relation with the use of different resources.

A possible outcome of our technique, i.e. determining how foraging behaviour could influence life-history traits (Figure 1), may be achieved by using existing methods for sampling individual foraging behaviour (as discussed later) and life-history traits. This component is critical to scale-up from individual foraging behaviour to processes occurring at the population scale. This can be performed by monitoring, for instance, survival rates, reproductive success, number and mass of offspring, individual mass or growth rate during or at the end of the foraging behaviour study period. In our study, we sampled critical life-history traits of large temperate herbivores [25], [26], [27] both simultaneously (winter survival) and successively (female and foetus body mass at the onset of spring) to the foraging behaviour study period.

The probability of recovery of dyed faeces along networks and paths was low in our study (0.33 and 0.15, respectively) because conspecifics used the same tracks. Sampling individual foraging behaviour with our technique was therefore only possible along single tracks in this study population. This conclusion might be limited by the small sample size of single track segments sampled in our study (n = 13 segments). We remain confident, however, that with a larger sample size, we would have found similar results because GPS collars had a very high accuracy (mean = 4.3 m, 95% CI [1.7, 6.9]), thus reducing the probability that single tracks located within that buffer may have been used by conspecifics.

Our technique was only reliable for single tracks in our study. This could generate a bias if foraging behaviour differs between track types. For example, preferred resources might attract higher densities of individuals compared to less preferred ones. Thus, consumption of preferred resources could be more frequent on networks than on single tracks, where foraging would be biased towards less preferred resources. In our study system, the main facet of foraging behaviour studied was individual diet derived from faeces collected on single tracks. Diet estimates derived from those faeces are not biased towards resources consumed only on single tracks, because those faeces contained resources consumed over the range of track types used during the past days [28], [29]. While interpreting results from the sampling of fine-scale selection or movement rates, we will take into account this potential bias by restricting our conclusions to foraging behaviour as described on single tracks.

### Add-ons to Existing Methods for Sampling Foraging Behaviour

Many sampling techniques exist to assess foraging behaviour, yet most techniques have constraints that can limit their applicability for studying processes at the individual level over specific spatio-temporal scales. Behavioural observations are probably the most direct and exhaustive technique to assess foraging behaviour because they can provide an assessment of the entire foraging process, i.e. how individuals search for, acquire and consume food resources. This technique, however, has limited applicability in secretive species, remote areas or in species living in habitats where observations from a distance are impaired by the lack of visibility (e.g. forests; [13]). To overcome these constraints of working with forested secretive species, researchers have conducted detailed behavioural observations on hand-raised cervids [30], [31]. Our technique overcomes these constraints by remotely monitoring free-ranging individuals, using their tracks to identify precise foraging locations, and using biomarking to rule-out the possibility that single tracks found in the vicinity of GPS-locations could have been used by conspecifics.

Genetic identification of individuals from faecal samples [32], [33] could also be used to rule-out the possibility that signs of presence associated to a collared individual could have been left by conspecifics. This method would allow the validation of individual signs of presence during the entire study period and would thus extend the restricted window of validation of our technique (i.e. the effective marking period). In addition, faecal samples found around signs of presence at sites potentially used by conspecifics (e.g. networks of tracks and paths in our study) could be genotyped, providing additional samples to describe individual diet. Despite increasing efficiency and decreasing costs of genetic identification of individuals from faecal samples, such technique remains more costly than our approach in studies when telemetry is required anyway. For instance, telemetry monitoring is required in most studies that aim at linking foraging behaviour to life-history traits as it allows the assessment of life-history traits during and after the foraging study period. In addition, the degrading effect of weather on DNA decreases genotyping power [34], [35]. A robust protocol to detect genotyping errors also needs to be developed to avoid spurious results [32], [33], [35]. Finally, in the case of cervids, the high number of pellets found on tracks requires a sampling protocol designed to reduce the number of pellets analysed without introducing any sampling bias [32].

Stable isotope and fatty acid analyses are two relatively recent techniques designed to trace the contribution of food sources to the diet of individuals over a range of temporal windows, using chemical analyses of animal tissues [12], [36]. Despite all their advantages, these techniques hinge on a key requirement that is not met in many studies including ours, namely differences in isotopic or fatty acids composition among food sources. To characterise diet over a temporal scale similar to those assessed by stable isotope and fatty acid analyses, on can repeat faeces collection over the temporal scale of interest on the validated type of tracks or locations. The combined use of telemetry tracking and biomarking allowed us to overcome a main limitation of faecal analysis, i.e. that the collection of faeces from a known individual is not always possible in free-ranging populations [13], while avoiding the costs and time associated with developing and using an efficient genetic identification protocol.

### Guidelines on the Use of this Technique in Other Study Systems

**Figure 3:**
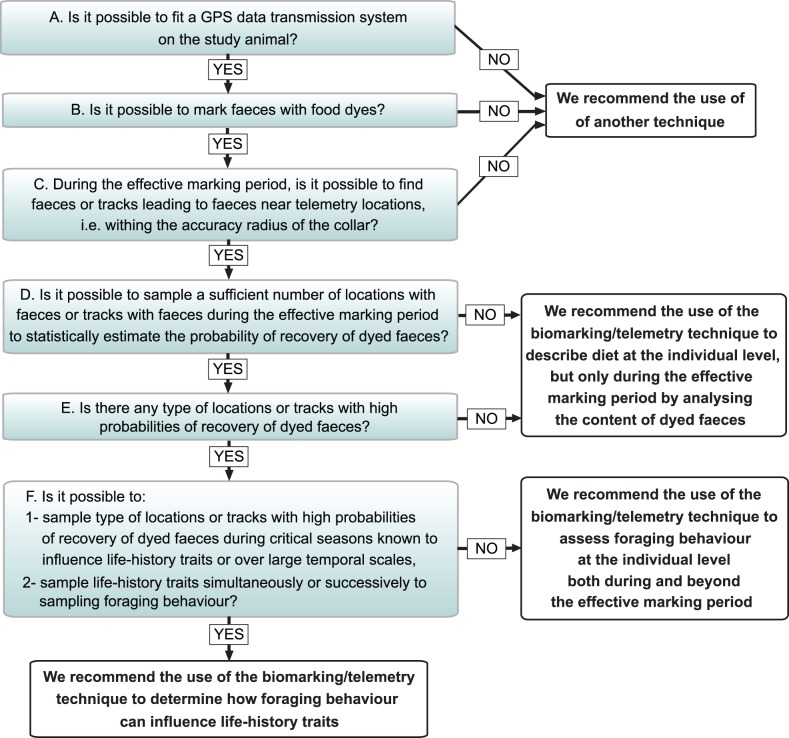
Decision tree for determining whether the biomarking/telemetry technique could be used in a given study system and which type of questions could be investigated.

We here provide guidelines for determining whether this technique could be used in a given study system and which type of questions could be investigated (see also the decision tree in Figure 3).

A) Determine the possible use of GPS data transmission systems on the study animals. Our technique requires to monitor the movements of animals remotely and to receive locations rapidly to avoid the loss of individual signs of animal presence through time. Here, we used GPS collars equipped with an Iridium satellite transmission device receiveing a high number of locations by email shortly after their recording (within 1–24 hours in our study). GPS collars equipped with other data transmission systems can also be used.

B) Determine the possible use of food dyes to mark animal faeces. We recommend verifying the effectiveness of different food dyes at different doses with the study species in captivity before using it in the wild. We also recommend using the captivity experiment to estimate the length of the effective marking period. In addition to FD&C Blue No. 1, dyes of other colours have been shown to stained ruminant faeces [37], or urine and faeces of small mammals and lagomorphs [38]. The fluorescent marker Rhodamine B was used to mark faeces and urine in lagomorphs [39], while glass beads marked faeces of canids and collared peccaries [40].

C) Determine the likelihood of recovering faeces or tracks leading to faeces near telemetry locations, within the accuracy radius of the GPS collar and during the effective marking period. In our study, we used snow tracks but faeces could be found near locations or on tracks left in other soft substrates such as mud, sand, or soft soil.

D) Determine the likelihood of sampling a sufficient number of tracks or locations with faeces during the effective marking period to statistically estimate the probability of recovering only dyed faeces. This would vary with the variability in the recovery of dyed faeces per type of locations or tracks, the length of the effective marking period, the frequency of occurrence of faeces near locations or on tracks, the number of individuals marked, and the speed of the GPS data transmission system. If such a sufficient sampling is not possible, our technique could still be used to describe individual diet during the effective marking period by analysing content of dyed faeces.

E) Determine whether there is any type of locations or tracks with a high probability of recovery of dyed faeces. If there are such locations or tracks, then sampling them could allow assessing foraging behaviour following our approach, both during and outside the effective marking period. To maximize the probability of validating locations or tracks, it is critical to use collars that are accurate enough to isolate individual signs of presence in a given study system. Higher accuracy will be required in study systems with higher spatial overlap between individuals. As accuracy of GPS collars in ecological studies generally ranges from 3 to 30 m and can vary between brands and models [19], we recommend chosing a collar brand and model matching the accuracy-related constraints of the study system (i.e. canopy closure and topography [19], as well as the degree of spatial overlap between individuals).

F) Determine the likelihood of sampling foraging behaviour over large temporal scales or during critical seasons known to influence life-history traits, and of sampling those traits during or at the end of sampling periods.

In conclusion, our sampling technique can be added in the toolbox of ecologists aiming at assessing individual foraging behaviour in secretive species, and moreover at ultimately investigating the links between foraging behaviour and life-history traits. This technique will contribute to a better understanding of how inter-individual variation within animal populations can shape demographic processes, and ultimately provide insights on how individual heterogeneity may drive more complex ecological processes.

## Supporting Information

Figure S1 **Probability of recovery of dyed faeces by track type.** Open circles represent raw data and closed squares represent predicted values with their 95% confidence intervals.
(DOCX)

Table S1 **Detailed results of model selection for mixed logistic regressions fitted to estimate the probability of sampling only dyed faeces for the different types of tracks (n = 96).** We took into account the non-independence of data from the same trail (random variable: trail ID). We modelled the effects of track type (Type), occurrence of snowfall (Snow), time of sampling during the day (Time), time elapsed since the capture (Capture-Trail), as well as biologically relevant interactions (i.e. Type × Snow, and Type × Time). We report the number of parameters (*k*), the Akaike information criterion for small sample sizes (AICc) relative to the model with the lowest AICc (ΔAICc), as well as the AICc weight (ωAICc). Models are ranked by their AICc values and the best model is shown in bold.
(DOCX)

Methods S1 **Estimation of collar accuracy.**
(DOCX)
