# Supplementary material for: A New Way of Assessing Foraging Behaviour at the Individual Level Using Faeces Marking and Satellite Telemetry
Source: PLoS One. 2012 Nov 16;7(11):e49719. doi: 10.1371/journal.pone.0049719 (PMC3500326; doi:10.1371/journal.pone.0049719)

**Figure S1. Probability of recovery of dyed faeces by track type.** Open circles represent raw data and closed squares represent predicted values with their 95% confidence intervals.


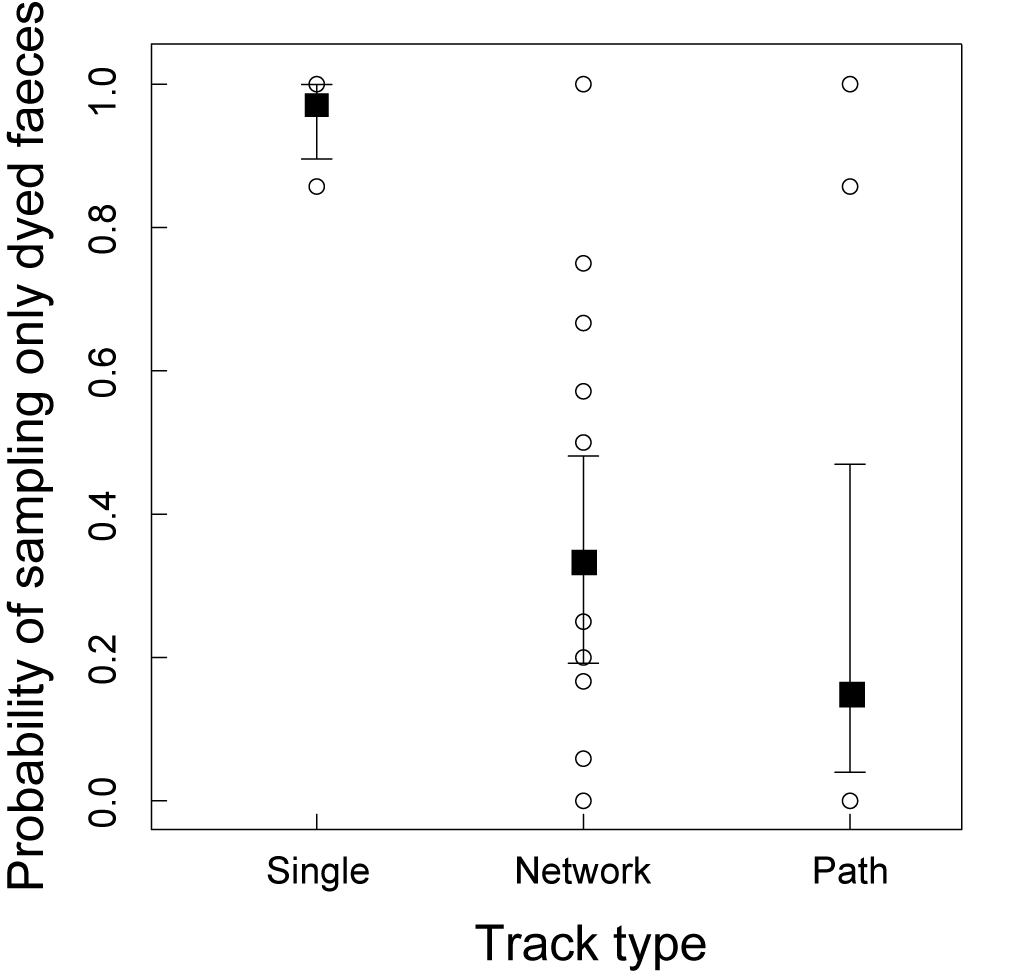

Supplement: Figure S1 — Probability of recovery of dyed faeces by track type. Open circles represent raw data and closed squares represent predicted values with their 95% confidence intervals. (DOCX) [file pone.0049719.s001.docx]
