# Supplementary material for: A New Way of Assessing Foraging Behaviour at the Individual Level Using Faeces Marking and Satellite Telemetry
Source: PLoS One. 2012 Nov 16;7(11):e49719. doi: 10.1371/journal.pone.0049719 (PMC3500326; doi:10.1371/journal.pone.0049719)
Supplement: Table S1 — Detailed results of model selection for mixed logistic regressions fitted to estimate the probability of sampling only dyed faeces for the different types of tracks (n = 96). We took into account the non-independence of data from the same trail (random variable: trail ID). We modelled the effects of track type (Type), occurrence of snowfall (Snow), time of sampling during the day (Time), time elapsed since the capture (Capture-Trail), as well as biologically relevant interactions (i.e. Type × Snow, and Type × Time). We report the number of parameters (k), the Akaike information criterion for small sample sizes (AICc) relative to the model with the lowest AICc (ΔAICc), as well as the AICc weight (ωAICc). Models are ranked by their AICc values and the best model is shown in bold. (DOCX) [file pone.0049719.s002.docx]

**Table S1. Detailed results of model selection for mixed logistic regressions fitted to estimate the probability of sampling only dyed faeces for the different types of tracks (n=96).** We took into account the non-independence of data from the same trail (random variable: trail ID). We modelled the effects of track type (Type), occurrence of snowfall (Snow), time of sampling during the day (Time), time elapsed since the capture (Capture-Trail), as well as biologically relevant interactions (i.e. Type × Snow, and Type × Time). We report the number of parameters (*k*), the Akaike information criterion for small sample sizes (AICc) relative to the model with the lowest AICc (ΔAICc), as well as the AICc weight (ωAICc). Models are ranked by their AICc values and the best model is shown in bold.

| Models | *k* | AICc | ΔAICc | ωAICc |
| --- | --- | --- | --- | --- |
| **Type** | **4** | **160.1** | **0.0** | **0.27** |
| Type+Snow | 5 | 161.4 | 1.3 | 0.14 |
| Type+Time | 5 | 161.6 | 1.5 | 0.13 |
| Type+Capture-Trail | 5 | 162.2 | 2.2 | 0.09 |
| Type × Snow | 7 | 162.3 | 2.2 | 0.09 |
| Type+Snow+Time | 6 | 163.2 | 3.1 | 0.06 |
| Type+Snow+Capture-Trail | 6 | 163.6 | 3.5 | 0.05 |
| Type+Time+Capture-Trail | 6 | 163.8 | 3.7 | 0.04 |
| Type × Snow+Time | 8 | 164.4 | 4.4 | 0.03 |
| Type × Snow+Capture-Trail | 8 | 164.5 | 4.5 | 0.03 |
| Type × Time | 7 | 165.3 | 5.2 | 0.02 |
| Type+Snow+Time+Capture-Trail | 7 | 165.5 | 5.5 | 0.02 |
| Type × Time+Snow+Capture-Trail | 9 | 166.8 | 6.7 | 0.01 |
| Type × Time+Capture-Trail | 8 | 167.2 | 7.1 | 0.01 |
| Type × Snow+Time+Capture-Trail | 8 | 167.6 | 7.6 | 0.01 |
| Type × Snow+Type × Time+Capture-Trail | 10 | 169.3 | 9.3 | 0.00 |
| Type × Snow+Type × Time | 9 | 169.6 | 9.5 | 0.00 |
| Type × Time+Snow | 11 | 171.8 | 11.7 | 0.00 |
| Null model | 2 | 192.3 | 32.3 | 0.00 |
