# Supplementary material for: A New Way of Assessing Foraging Behaviour at the Individual Level Using Faeces Marking and Satellite Telemetry
Source: PLoS One. 2012 Nov 16;7(11):e49719. doi: 10.1371/journal.pone.0049719 (PMC3500326; doi:10.1371/journal.pone.0049719)
Supplement: Methods S1 — Estimation of collar accuracy. (DOCX) [file pone.0049719.s003.docx]

### **Methods S1. Estimation of collar accuracy.**

We estimated collar accuracy in two ways. First, we measured the average distance between locations recorded by stationary paired GPS collars and handheld GPSs in both open and forest habitats [[1](#_ENREF_1)]. We used 15 pairs of handheld GPS and collars in an open habitat (clear-cut) and 14 pairs in a forest habitat (white-spruce [*Picea glauca*] stand). We selected this method because it mimics our field monitoring protocol where we used handheld GPS to find locations transmitted by collars. We also estimated accuracy by averaging the distance between each location recorded by a stationary GPS collar and the average location recorded by the same collar over 48h, using 15 and 14 collars in open and forest habitat types, respectively. We used this second method to provide estimates that can be compared with other studies using this method [[2](#_ENREF_2)].

For both approaches, we verified if estimated accuracy differed between habitat types using a linear model performed with the function lm in R. We did not detect a statistical difference in the accuracy of collars between open and closed habitat types for both approaches, and including or excluding two outliers that had high leverage values did not change those results. When comparing locations recorded by paired collars and handheld GPS, the accuracy predicted from the model was 5.0 m (95% CI = [1.6, 8.4]) in the clear-cut and 3.3 m (95% CI = [0.0, 6.9]) in the white spruce stand. When comparing each location recorded by collars with the average of all locations per collar, the accuracy predicted from the model was 4.8 m (95% CI = [3.8, 6.6]) in the clear-cut and 5.2 m (95% CI = [3.8, 6.6]) in the white spruce stand.

Because there was no difference in accuracy between open and closed habitat types, we computed the average accuracy of collars using all data from both habitat types (first approach: 4.3 m, 95% CI = [1.7, 6.9]; second approach: 5.0, 95% CI = [4.0, 5.9]). Both approaches gave similar results, so we retained the estimates obtained with the first approach as the confidence intervals are more conservatives, and as it better mimics our field monitoring protocol.

**References**

1. Hansen MC, Riggs RA (2008) Accuracy, precision, and observation rates of global positioning system telemetry collars. Journal of Wildlife Management 72: 518-526.

2. Frair JL, Fieberg J, Hebblewhite M, Cagnacci F, DeCesare NJ, et al. (2010) Resolving issues of imprecise and habitat-biased locations in ecological analyses using GPS telemetry data. Philosophical Transactions of the Royal Society B: Biological Sciences 365: 2187-2200.
